# Bacterial Nanocellulose and Its Surface Modification by Glycidyl Methacrylate and Ethylene Glycol Dimethacrylate. Incorporation of Vancomycin and Ciprofloxacin

**DOI:** 10.3390/nano9121668

**Published:** 2019-11-22

**Authors:** Elena Vismara, Andrea Bernardi, Chiara Bongio, Silvia Farè, Salvatore Pappalardo, Andrea Serafini, Loredano Pollegioni, Elena Rosini, Giangiacomo Torri

**Affiliations:** 1 Department of Chemistry, Materials and Chemical Engineering “Giulio Natta”, Politecnico di Milano, via Mancinelli 7, 20131 Milano, Italy; andrea.bernardi@polimi.it (A.B.); chiara.bongio@polimi.it (C.B.); silvia.fare@polimi.it (S.F.); salvatore.pappalardo@polimi.it (S.P.); andrea.serafini@polimi.it (A.S.); 2 Department of Biotechnology and Life Sciences, Università degli Studi dell’Insubria, via J.H. Dunant 3, 21100 Varese, Italy; loredano.pollegioni@uninsubria.it (L.P.); elena.rosini@uninsubria.it (E.R.); 3 Istituto Scientifico di Chimica e Biochimica “Giuliana Ronzoni”, via Giuseppe Colombo 81, 20133 Milano, Italy; torri@ronzoni.it

**Keywords:** bacterial nanocellulose, methacrylate, Fenton reagent, cross-linking, vancomycin, ciprofloxacin, bioactive bacterial nanocellulose

## Abstract

Among nanocelluloses, bacterial nanocellulose (BNC) has proven to be a promising candidate in a range of biomedical applications, from topical wound dressings to tissue-engineering scaffolds. Chemical modifications and incorporation of bioactive molecules have been obtained, further increasing the potential of BNC. This study describes the incorporation of vancomycin and ciprofloxacin in BNC and in modified BNC to afford bioactive BNCs suitable for topical wound dressings and tissue-engineering scaffolds. BNC was modified by grafting glycidylmethacrylate (GMA) and further cross-linking with ethylene glycol dimethacrylate (EGDMA) with the formation of stable C–C bonds through a radical Fenton-type process that involves generation of cellulose carbon centred radicals scavenged by methacrylate structures. The average molar substitution degree MS (MS = methacrylate residue per glucose unit, measured by Fourier transform infrared (FT–IR) analysis) can be modulated in a large range from 0.1 up to 3. BNC-GMA, BNC-EGDMA and BNC-GMA-EGDMA maintain the hydrogel status until MS reaches the value of 1. The mechanical stress resistance increase of BNC-GMA and BNC-GMA-EGDMA of MS around 0.8 with respect to BNC suggests that they can be preferred to BNC for tissue-engineering scaffolds in cases where the resistance plays a crucial role. BNC, BNC-GMA, BNC-EGDMA and BNC-GMA-EGDMA were loaded with vancomycin (VC) and ciprofloxacin (CP) and submitted to release experiments. BNC-GMA-EGDMA of high substitution degree (0.7–1) hold up to 50 percentage of the loaded vancomycin and ciprofloxacin amount, suggesting that they can be further investigated for long-term antimicrobial activity. Furthermore, they were not colonized by *Staphylococcus aureus* (S.A.) and *Klebsiella pneumonia* (K.P.). Grafting and cross-linking BNC modification emerges from our results as a good choice to improve the BNC potential in biomedical applications like topical wound dressings and tissue-engineering scaffolds.

## 1. Introduction

Nanocelluloses (NCs), namely cellulose-based materials with peculiar physicochemical properties, appear as a new option offering a wide range of specific applications quite different from cellulose. Nanocellulose-based biomaterials have been significantly investigated for biomedical applications due to their excellent physical and biological properties like biocompatibility and low cytotoxicity [1].

A nanocellulose that has been studied in recent years is bacterial nanocellulose (BNC), a nanofibrilar polymer produced by several species of bacteria [2]. BNC is a hydrogel containing 1% of nanocellulose. It cannot be ignored by researchers interested in nanocellulose due to its unique properties, such as chemical purity, biocompatibility, inertness and non-toxicity, biofunctionality and hypoalergenicity, good mechanical strength, high absorbency, and the possibility of forming any shape and size. Due to its properties, the study and use of BNC are focused on biomedicine. Tissue engineering has been associated with BNC mostly because of its low cytotoxicity, high porosity, biocompatibility, and non-resorbability [1]. For soft-tissue implants and cartilage replacements, the fibrillar network of BNC offers high tensile mechanical properties and a hydrogel-like behaviour as BNC interacts with the surrounding water medium. However, despite recent advances, there are still many challenges to overcome before the full potential of BNC can be completely realized as a choice of material in tissue-engineering applications. Different physical modification, i.e., modification to change porosities, crystallinities, and fibre densities, and chemical modifications, i.e., modification of the chemical structures and functionalities, have been investigated to enhance BNC properties suitable for its applications in tissue-engineering applications [3].

In the recent review on nanocellulose as a natural source for groundbreaking applications in materials science, BNC’s future has been widely discussed [4]. The key problems seem to be BNC bioreactor designs and implant production costs. Economic considerations related to different applications must be taken into account: a high-price implant material, for example, in a first artificial heart bypass, can be more competitive than a Nata de Coco-based food thickener obtained in mass production. The conclusion of authors of reference [4] is that nowadays there are no standard answers regarding BNC production costs. Noteworthy, some BNC-based medical products are on the market and are Food and Drug Administration (FDA) approved and European Community (CE) -certified.

Besides the efforts dedicated to the advancement in production technologies and the estimation of various pitfalls associated with BNC production, a more scientific question is intriguing, if BNC and its composites with other polymers/nanoparticles can become the material of preference in current regenerative medicine, starting from biomedical applications of BNC and functionalized BNC in drug delivery, tissue engineering and antimicrobial wound healing [5]. A very recent paper stating the BNC haemocompatibility favours developing BNC biomedical applications [6]. Furthermore, from a chemical point of view, BNC is chemically identical with plant cellulose but is free of byproducts like lignin, pectin, and hemicelluloses, featuring a unique reticulate network of fine fibres and these are physicochemical peculiarities that make the difference between BNC and other nanocellulose materials [7]. BNC has been already been proposed as a skin-tissue repair material in vivo to replace conventional gauze dressings. According to the results of many studies in the field of wound healing, BNC has been shown to be a superior candidate for conventional wound-dressing materials [8]. For tissue engineering and wound healing, the problems of infection and inflammation have to be carefully considered as BNC does not possess any antimicrobial or anti-inflammatory characteristics. BNC can be endowed with antimicrobial or anti-inflammatory activities by drug loading. Drug loading in BNC-based carriers includes physical absorption/adsorption and chemical conjugation [9]. BNC hydrogels can be fabricated using as low as 1% nanocelluloses, which may benefit from a large water content, that allows for the drugs to be loaded via diffusion and adsorption; however, the downside of this process is the prolonged loading time, which is not practical for clinical or industrial purposes. The loading time is a minimum 24 h and can reach 48 h. As the amount of the loaded drug is evaluated by the release experiments it is difficult to measure the real adsorbed amount. 

Anyway, the most common loading method involves the immersion of the BNC delivery system in the drug solution. For example, BNC has been saturated with the antibiotic fusidic acid [10] and tetracycline hydrochloride has been loaded on bacterial cellulose composite membranes for controlled release [11]. BNC can be addressed for the delivery of a broad range of bioactive cargos [9]. The antiseptic octenidine has been used to provide active wound dressings based on bacterial nanocellulose [12]. The behaviour of BNC membranes has been studied as systems for topical delivery of lidocaine, an anaesthetic drug with high solubility in water in the form of hydrochloride and commonly used in surgery and topical application [13]. 

Since 2009 a review described the design and applications of biodegradable cellulose-based hydrogels and suggested that cellulose-based hydrogels, including BNC, could be ideal platforms for the design of scaffolding biomaterials in the field of tissue engineering and regenerative medicine [14]. The advantage of BNC is that it is obtained by a strain directly in the hydrogel status. Hydrogel-based devices have been developed for controlled drug delivery. 

The aim of this study is to provide bioactive BNCs in the form of hydrogel, suitable for tissue engineering, regenerative medicine and wound dressing. We focus our attention on modified BNCs, in particular grafted and cross-linked BNC, made bioactive by loading drugs. The study concerns also the loading of BNC as it is and the comparison between BNC and modified BNC. The modifications were pursued by grafting glycidylmethacrylate (GMA) and cross-linking with ethylene glycol dimethacrylate (EGDMA). GMA-grafted and EGDMA-cross-linked BNCs of peculiar chemical and mechanical properties were obtained by a surface modification that maintained the original network structure of BNC. As concerns this last aspect, our study can be positioned in a relationship with BNC nanocomposites processing techniques that allow the incorporation of functional nanoreinforcements, nanofillers and additional phases without disturbing the original network structure of BNC [15]. The choice of GMA was justified by previous results that stated that GMA appendages endow cellulose with new properties due to the surface GMA network formation that acts like a molecular sponge [16]. GMA insertion is a radical-based process that was investigated in term of mechanism, detailed in all the synthetic aspects and huge supported by different characterisation techniques. Cellulose maintained its peculiar properties, as the methodology to insert GMA did not induce a coating by avoiding its polymerisation. GMA-modified cellulose was defined as a multitasking material as it found applications in quite different fields [16]. The adsorption and release properties of antibiotics is strictly related to the aim of this paper [17]. GMA-grafted fabrics loaded with vancomycin and other antibiotics were developed for topic antibacterial activity [18]. For BNC modification, GMA was also associated to EGDMA, a very efficient cross-linking agent already tested on nanocellulose [19] and on imprinted hydrogel prepared from *N*,*N*-dimethylacrylamide and tris(trimethylsiloxy)sililpropyl methacrylate as the main components, methacrylic acid as functional monomer [20]. The idea of using EGDMA comes not only from the need to reinforce BNC, but also from the hope that GMA and EGDMA can act in synergy in holding drugs. BNC and GMA-grafted and EGDMA-cross-linked BNCs were made bioactive by loading the antibiotics vancomycin and ciprofloxacin. Vancomycin is a glycopeptide antibiotic used in the treatment of infections caused by Gram-positive bacteria, such as *Staphylococcus aureus*. Vancomycin has been found many applications for bone regeneration. Notably, it does not interfere with normal in vivo bone regeneration [21]. It has also been selected as an antibiotic drug for enhanced bone regeneration [22]. Finally vancomycin has been used as polyhydroxy antibiotic for bio-inspired cross-linking and matrix–drug interactions for advanced wound dressings with long-term antimicrobial activity [23]. The broad-spectrum antibiotic ciprofloxacin has been used as model drug for BCN-cyclodextrin adduct to form drug-nanocarrier systems [24].

## 2. Materials and Methods

Hydrogen peroxide (H_2_O_2_) solution 30% (*w*/*w*), glycidyl methacrylate (GMA), ethylene glycol dimethacrylate (EGDM), iron sulfate heptahydrate (FeSO_4_∙7H_2_O), sodium hydroxide (NaOH), hydrochloric acid (HCl, 36.0–38.0% in water) and corn step liquor were purchased from Sigma Aldrich (St. Louis, MO, USA). *Gluconacetobacter xylinus* (ATCC 10245) was purchased from the American Type Culture Collection (ATCC, Manassas, VA, USA).

### 2.1. Synthesis of Bacterial Nanocellulose (BNC)

BNC was obtained by the use of the bacterium *Gluconacetobacter xylinus* (ATCC 10245). The microorganism was inoculated in 5 mL ATCC medium 1 (5 g/L yeast extract, 3 g/L peptone, 25 g/L mannitol) and grown at 26 °C under static conditions for 48 h. An aliquot of 1 mL of this culture was transferred in a 250 mL Erlenmeyer flask containing 40 mL of corn steep liquor (CSL)-glucose medium (26 g/L CSL, 20 g/L glucose, 2.7 g/L Na_2_HPO_4_, 1.15 g/L citric acid monohydrate). The inoculated flasks were incubated at 30 °C for 144 h under static conditions. The BNC disk was removed from the medium and washed exhaustively with distilled water. Overnight treatment with 70 mL NaOH 1M followed by washing with water until neutral pH bleached the BNC sample. The final weight was 2.56 g.

### 2.2. Grafting of BNC

BNC was put in a three-neck round-bottom flask equipped with a thermometer and a condenser, containing 25 mL water for 1 g of BNC, reaction scale and reagent amount being reported in Table 1 and Table 2. The suspension was maintained under mechanic stirring at 80 °C for 30 min. H_2_O_2_ and FeSO_4_ (Fenton reagent) were added and left activating cellulose for 1 h at 80 °C. GMA was dropped in the flask and left to react for 30 min. BNC was filtered using a Gooch funnel and exhaustively washed with water at room temperature and with water at 45 °C to remove residual inorganic and organic reagents. The washed BNC was stored at 8 °C.

### 2.3. Grafting and Cross-Linking of BNC

BNC was put in a three-neck round-bottom flask equipped with a thermometer and a condenser, containing 50 mL water for 2.2 g of BNC, reaction scale and reagent amount being reported in Table 3. The suspension was maintained under mechanical stirring at 80 °C for 30 min. H_2_O_2_ and FeSO_4_ (Fenton reagent) and left activating cellulose for 1 h at 80 °C. GMA and EGDMA were simultaneously dropped in the flask and left reacting for 30 min. BNC was filtered using a Gooch funnel and exhaustively washed with water at room temperature and with water at 45 °C to remove residual inorganic and organic reagents. The washed BNC was stored at 8 °C.

### 2.4. Fourier Transform Infrared (FT–IR) Spectroscopy Analysis

The solid phase Fourier transform infrared (FT–IR) spectra of the powdered sample, obtained by BNC lyophilization and mixed with infrared-grade KBr, were generated using an ALPHA spectrometer (Bruker, Bremen, Germany). Data were analyzed using OPUS software, version 7.0 (Bruker, Bremen, Germany). The acquisition of the spectra were performed in the range 4000–400 cm^−1^. The estimation of average molar substitution ratio (MS) was obtained with the method used in our previous works, Equation (1) [16,25]:

(1) $${\mathrm{MS}}_{\mathrm{FT}-\mathrm{IR}}=\frac{\mathrm{area}\;\mathrm{ester}\;\left(\mathrm{manual}\;\mathrm{band}\;\mathrm{integration}\right)}{\mathrm{area}\;\mathrm{cellulose}\;\left(\mathrm{range}\;780-465{\;\mathrm{cm}}^{-1}\;\mathrm{integration}\right)}$$

At least two tests were carried out to assure reproducibility and accuracy for the integration calculations. 

### 2.5. Solid-State Cross-Polarization Magic-Angle Spinning (CP-MAS) ^13^C Nuclear Magnetic Resonance (NMR)

The ^13^C nuclear magnetic resonance (NMR) analysis was performed by using a dipolar decoupling cross-polarization magic-angle spinning (DD-CP-MAS) technique with a Bruker Avance 300 spectrometer (75.47 MHz, Bruker, Milano, Italy). Concerning data acquisition parameters: the repetition time (D1) was equal to 8 s, while the contact time and the spin rate were 2 ms and 1000 Hz, respectively. 2 K scans were collected to obtain good quality spectra. The samples were positioned in a zirconium rotor (diameter: 4 mm; height: 21 mm). Tetramethylsilane was the reference substance for the chemical shifts. Benzene was used as secondary reference standard.

The crystallinity index (Cr. I %) of BNC materials was evaluated by means of Equation (2) [16,25]. *A* corresponds to integrals of the C-4 peaks at 86–92 ppm (crystalline) and *B* to the integrals of the C-4 peaks at 80–86 ppm (amorphous).

(2) $$\mathrm{Cr}.I\;\left(\%\right)=\frac{A}{A+B}\times 100$$

### 2.6. Scanning Electron Microscopy (SEM) Experiments

Scanning electron microscopy (SEM) micrographs of the film surfaces were obtained on a Zeiss EVO 50 microscope (Zeiss, New York, NY, USA), equipped with a LaB_6_ electron gun and operating at 15 kV. SEM was used to measure BNC fibre diameters and to study their morphologies. Prior to the SEM observation, a few nanometers gold film was deposited onto the sample surface in order to avoid a charging effect during SEM testing. The BNC fibers diameters statistical distributions were obtained analyzing the SEM micrographs with ImageJ (version 1.52r, NIH, National Institutes of Health, Bethesda, MD, USA) software [26], collecting more than 300 diameter sizes for each specimen. 

### 2.7. Mechanical Properties

The structure and the properties of BNC strongly depend on the choice of the cellulose forming bacterial strain. The tensile strength changes significantly, too. For *Gluconacetobacter xylinus* ATCC 10245 the tensile strength is reported 0.184 mPa ([2], p. 185). Tensile strength and elongation to failure of BNC samples of 2 cm length, 0.5 cm width and 0.5 thickness were measured with a Dynamic Mechanical Analyzer (model DMA Q800, TA instruments, Milano, Italy). Samples were air dried for 2 h, with a water reduction content of the 50%. The compression reduced the water content of the 80%.

### 2.8. Adsorption and Release Experiments

Aqueous solutions of vancomycin (VC) and ciprofloxacin (CP), having a concentration 2 × 10^−3^ M were prepared. The CP solution was at pH = 3 with HCl 0.02 N to solubilize CP. BNCs (1.5 g, solid nanocellulose content 15 mg) were put into contact with the drug solution (25 mL). The flask was shaken in a thermostatic bath Julabo at 25 °C, (model SW22, 100 rpm, JULABO GmbH, Seelbach, Germany) for 24 h to get the equilibrium. Adsorption solutions were analyzed at different times with an ultraviolet (UV) spectrophotometer. Loaded BNCs were submitted to release experiments by removing the BNC from the adsorption solution and transferring it into a flask with deionized pure water (25 mL). The amount of loaded drug release was evaluated by submitting at different times the release solution to UV spectrophotometry.

### 2.9. Ultraviolet (UV) Spectroscopy Analysis

The amount of adsorbed/released molecules over time was monitored through quantitative ultraviolet–visible (UV–Vis) analysis (UV-spectrophotometer JASCO V-650; Jasco Europe, Cremella LC, Italy), Spectra Manager^TM^ software (version II, Jasco Europe, Cremella LC, Italy). Several aliquots of drug solution were collected at different times and submitted to UV acquisition. The acquisition wavelength range was set from 500 to 240 nm. The absorbance values of VC peak at λ_max_ = 280 nm and CP peak at λ_max_ = 272 nm were converted in concentration by using previously created calibration curves.

### 2.10. Antibacterial Activity Tests 

The tests were run according to UNIEN ISO 20645:2005 by Centrocot (www.centrocot.it). *Staphylococcus aureus* (ATCC 6538 LOT: DSM 799-0415) and *Klebsiella pneumoniae* (ATCC 4352 LOT: DSM 789-0513) were cultured in Triptone and Soya medium for 24 h at 37 °C. The suspensions obtained were diluted to 10^8^ UFC/mL. 1 mL samples (10^8^ UFC/mL) were added to 150 mL of Triptone Soya Agar (LOT: Oxoid 1837431) at 45 °C to afford culture medium. A BNC sample of size 25 ± 5 mm was incubated in 5 mL of the culture medium for 24 h at 37 °C. The test was repeated on four equal BNC samples. Reference material was 100% cotton fabric according to International Organization for Standardization (ISO) 105/F02:2009. 

## 3. Results

The synthetic procedures were first developed and optimised. Both modified BNCs and BNC were exhaustively characterised. Finally modified BNCs and BNC as it is were loaded with the drugs. The loaded BNCs were submitted to release studies and to in vitro antibacterial activity tests. 

### 3.1. Grafting and Cross-Linking of BNC

**Figure 1:**
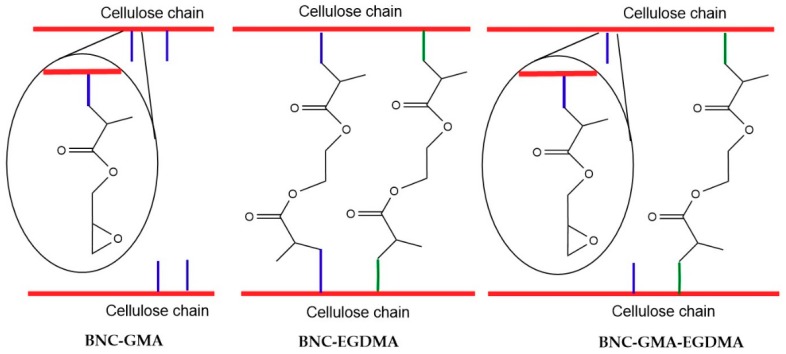
BNC-GMA (left); BNC-EGDMA (centre); BNC-GMA-EGDMA (right).

This process provides BNC-GMA, BNC-EGDMA and BNC-GMA-EGDMA, as shown by Figure 1.

**Scheme 1:**
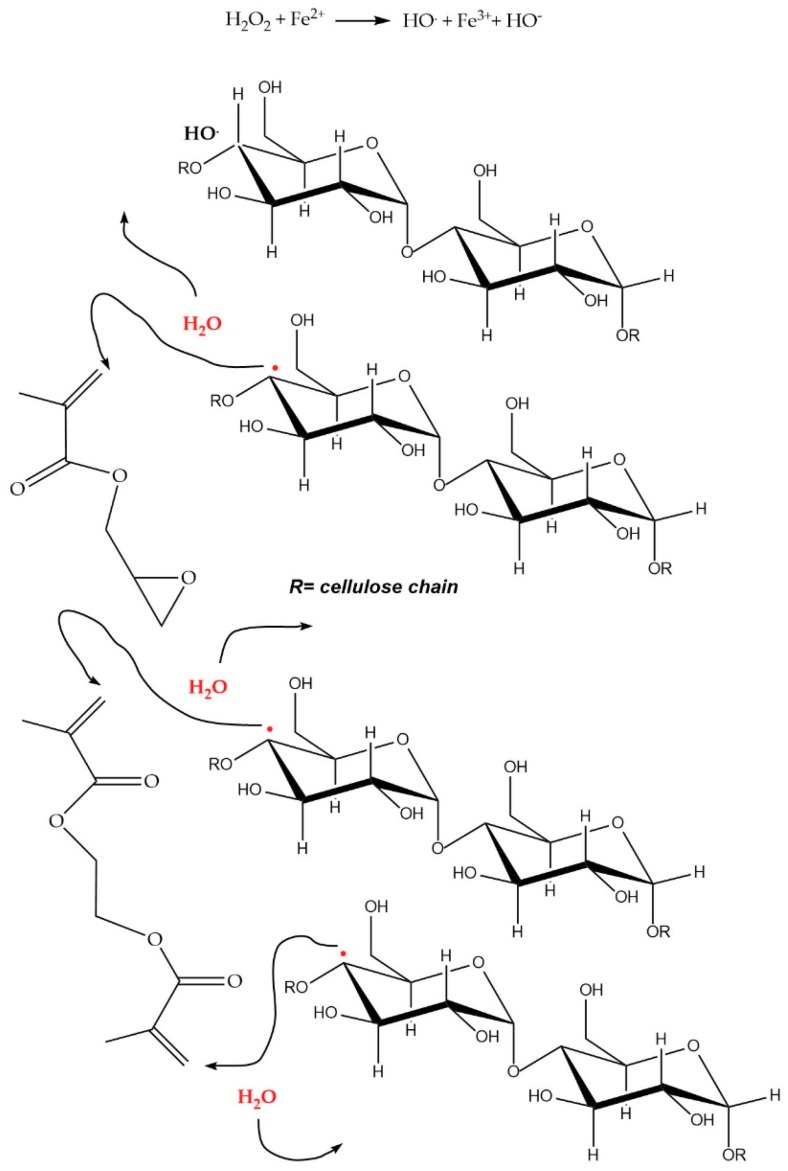
Grafting and cross-linking mechanism. Radical generation and trapping.

BNC was modified by the radical process that involves hydrogen peroxide and iron salt (Fenton reagent) generation of cellulose carbon centred radicals scavenged by glycidyl methacrylate (GMA) and by ethylene glycol dimethacrylate (EGDMA), see Scheme 1 [16,25].

As the hydrogen abstraction is unselective due to the high reactivity of OH, the grafting and cross-linking occurs randomly on the cellulose chain. For convenience, only C4 radical was put in evidence in the Scheme 1. GMA scavenging results in branching cellulose, while EGDMA scavenging results in cross-linking cellulose. To quantify the scavenging yields the average molar substitution degree (MS) was defined as the number of GMA/EGDMA residue per glucose unit and measured by FT–IR spectrum as detailed in materials and methods paragraph. In agreement with the random functionalization MS is an average measure. Nevertheless it is important to point out that with this approach cellulose is not coated by a methacrylate polymer, as shown by FT–IR and NMR spectra, but only functionalized. 

**Figure 2:**
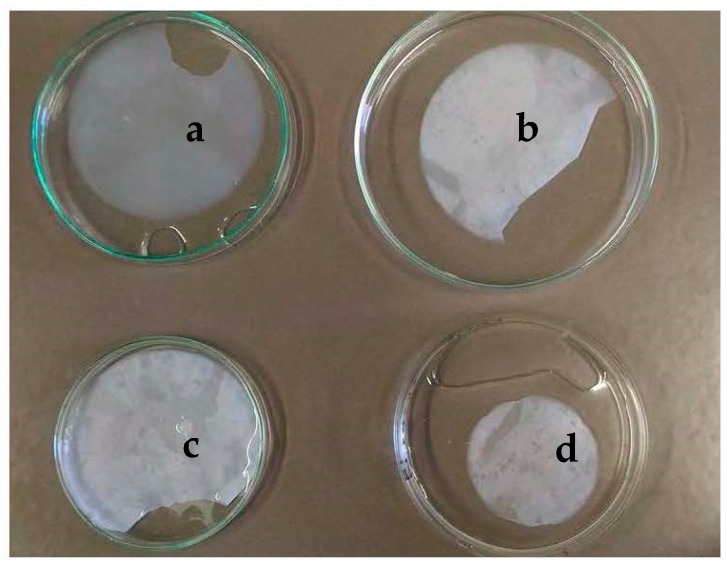
BNC (**a**); BNC-GMA MS = 0.25 (**b**); BNC-GMA-EGDMA MS = 0.8 (**c**); BNC-EGDMA MS = 0.55 (**d**).

Figure 2 is the picture of BNC and grafted and cross-linked BNC. The functionalization reduces the BNC transparency that appears whiter.

#### 3.1.1. Glycidylmethacrylate (GMA) Grafting

Table 1 reports the GMA grafting results on 390 mg of BNC containing 3.9 mg of nanocellulose (2.4 × 10^−2^ mmol of glucose unit) in function of the reagents amount. Every experiment was repeated at least three times. On this scale, the grafting reproducibility and homogeneity is very good. The molar ratios are normalized on 1 mmol of glucose unit (162 Da). 

**Figure 3:**
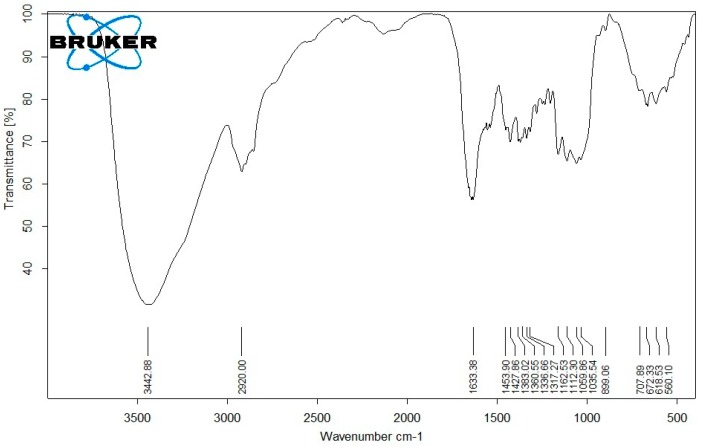
BNC Fourier transform infrared (FT–IR) spectrum: cellulose profile.

The BNC FT–IR spectrum can be seen in Figure 3.

**Figure 4:**
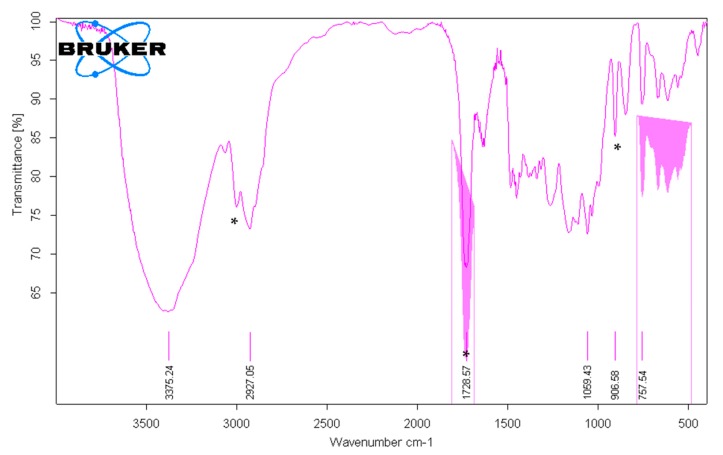
BNC-GMA MS = 0.8 FT–IR spectrum: cellulose profile *plus* GMA appendages (*).

The BNC-GMA MS = 0.8 FT–IR spectrum (Figure 4) confirms that the GMA grafting occurred by the appearance of the bands of the glycidyl ester peak signal at 1728.57 cm^−1^, of the C–H stretching signal of the epoxide ring signal at 3050–3000 cm^−1^ and of the C–O–C stretching signal of the epoxide ring at 1270 and 906.58 cm^−1^. In the spectrum of Figure 4 we put in evidence the epoxide bands (*), the band of the glycidyl ester (*) and the bands typical of the cellulose in the range 780–465 cm^−1^ (C–O–C, C–C–O, and C–C–H deformation and stretching vibrations), whose integrations ratio in Equation (1) is defined MS. 

**Figure 5:**
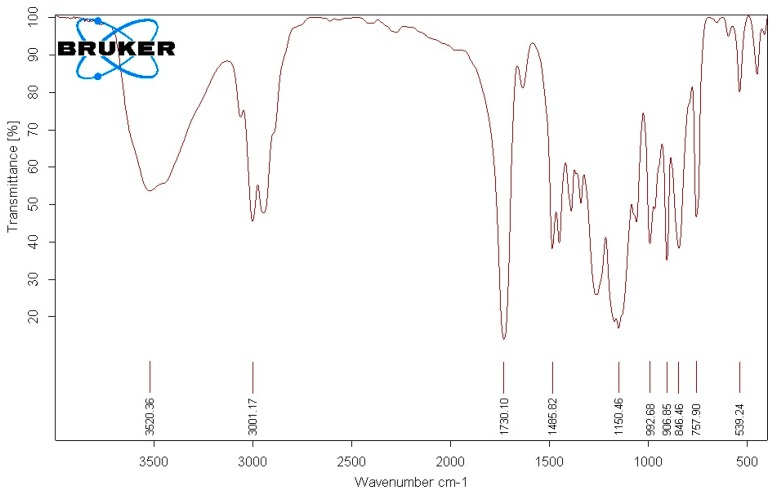
BNC-GMA MS = 1.8 FT–IR spectrum: cellulose profile *plus* GMA appendages.

In agreement with the reagents amount, see entries 3 and 4, Table 1, the BNC-GMA in entry 4, MS = 0.8, is less functionalized than the BNC-GMA in entry 3, MS = 1.8, as measured from their FT–IR spectra shown in Figure 4 and Figure 5, respectively.

Table 2 reports the GMA grafting on 2.1 g of BNC in function of the reagents amount.

**Figure 6:**
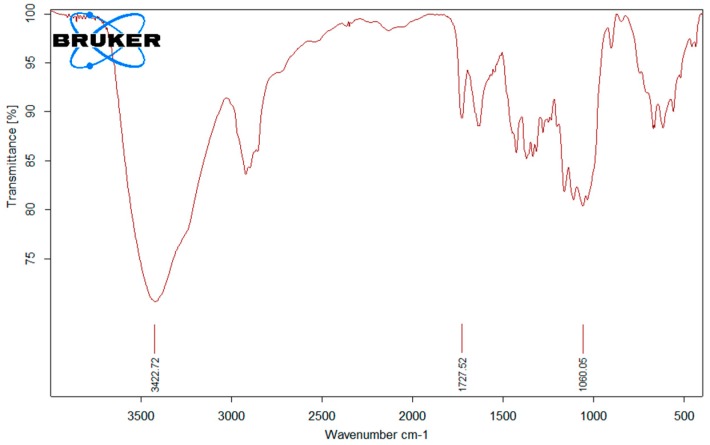
BNC-GMA MS = 0.12 FT–IR spectrum: cellulose profile *plus* GMA appendages.

When the reaction scale increases to more than 1 g, the grafting occurs with an acceptable reproducibility, being every experiment repeated at least three times, but it is not so easy to have homogeneous samples, as shown by the macroscopic aspect of the samples. Some zones appear more transparent than others, especially at low MS. Thus, every GMA BNC grafted sample was carefully mapped by FT–IR analysis that confirms the non-homogeneity. For convenience we report one spectrum, i.e., in Figure 6 the FT–IR spectrum of the less functionalized zone of MS = 0.12. In Table 2 the MS range calculated by FT–IR mapping is reported.

Data in Table 1 and Table 2 show that the GMA grafting can be modulated in a large range of MS. Until MS around 1, the hydrogel state was maintained. The water retention is little influenced by the functionalization. The percentage of cellulose measured by sample lyofilization changes from 1% for the starting BNC to 2–3% for MS around 0.6 and reaches 5% for MS around 1. The situation dramatically changed for highly functionalized BNC (MS > 2) that became solid and the water content was reduced to the half.

**Figure 7:**
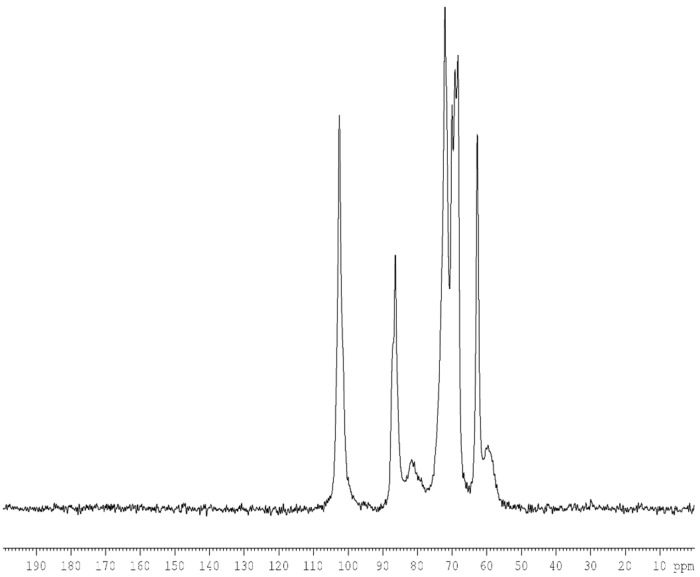
Cross-polarization magic-angle spinning (CP-MAS) ^13^C nuclear magnetic resonance (NMR) of BNC: cellulose signals.

**Figure 8:**
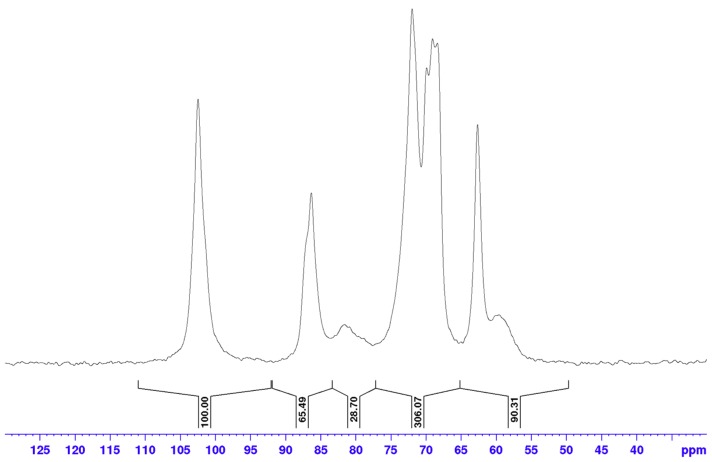
CP-MAS ^13^C NMR of BNC-integration.

CP-MAS ^13^C NMR spectra not only confirm the occurrence of the GMA grafting, but allow measurement of the crystallinity, see Equation (2). Figure 7 and Figure 8 show the BNC solid-state CP-MAS spectrum, the second one with the measure of the integrals used to calculate the Cr. I % = 69.

**Figure 9:**
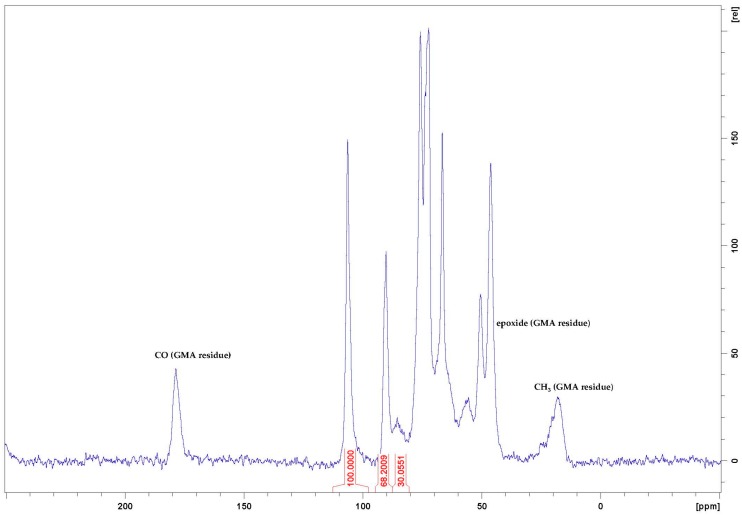
CP-MAS ^13^C NMR of BNC-GMA MS = 0.68 -integration: cellulose signals *plus* GMA signals.

Figure 9 shows the CP-MAS ^13^C NMR of BNC-GMA MS = 0.68 and its integration to measure Cr. I % = 69.

**Figure 10:**
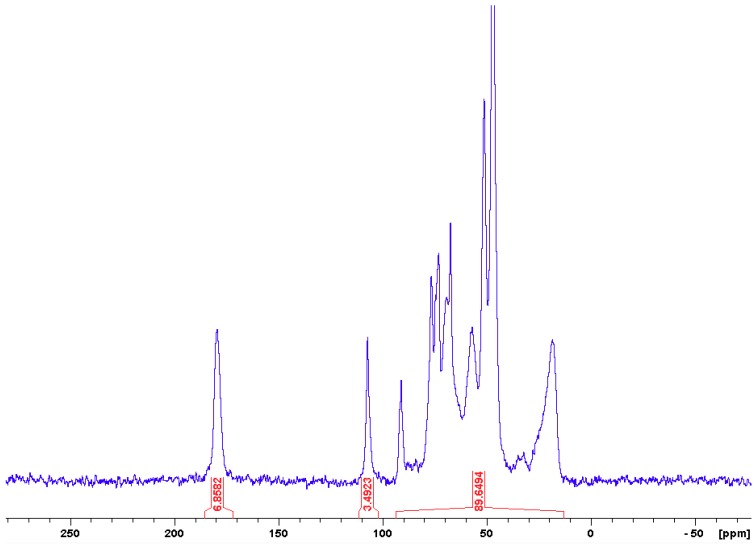
CP-MAS ^13^C NMR of BNC-GMA (MS >> 1)-integration Cr. I % ≈ 69: cellulose signals *plus* GMA signals.

Figure 10 shows that even highly functionalized BNC-GMA maintains the crystallinity. The conclusion is that GMA grafting does not modify the crystallinity of BNC as it is.

#### 3.1.2. GMA Grafting and Ethylene Glycol Dimethacrylate (EGDMA) Cross-Linking

The EGDMA cross-linking conditions were decided on the base of the results obtained for GMA grafting, as summarized in Table 3. GMA and EGDMA were put in the reactions at the same time in the molar ratio 2:1. EGDMA was also used alone, see entry 3. The quantitative data show that grafting/cross-linking seems more efficient than the only grafting, see entries 1 and 3, Table 3, compared with entry 2, Table 1. In entry 2, MS = 1 was reached by increasing the scavenging time from 30 min to 1 h.

**Figure 11:**
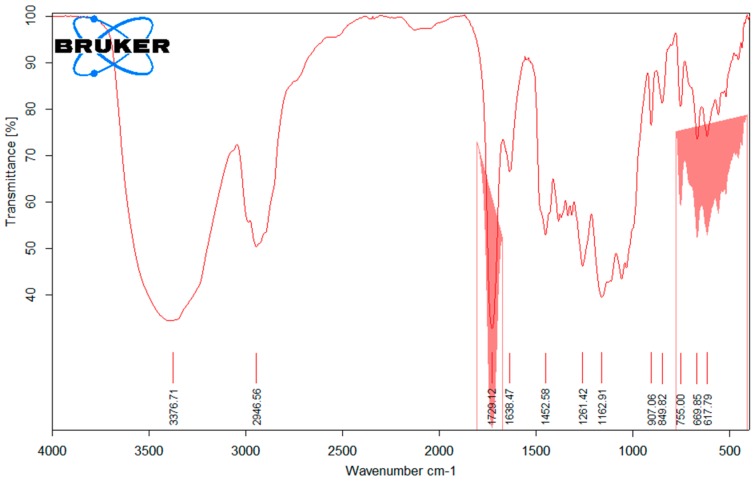
BNC-GMA-EGDMA MS = 0.6 FT–IR spectrum. GMA and EGDMA ester groups signal and cellulose reference signals in evidence.

**Figure 12:**
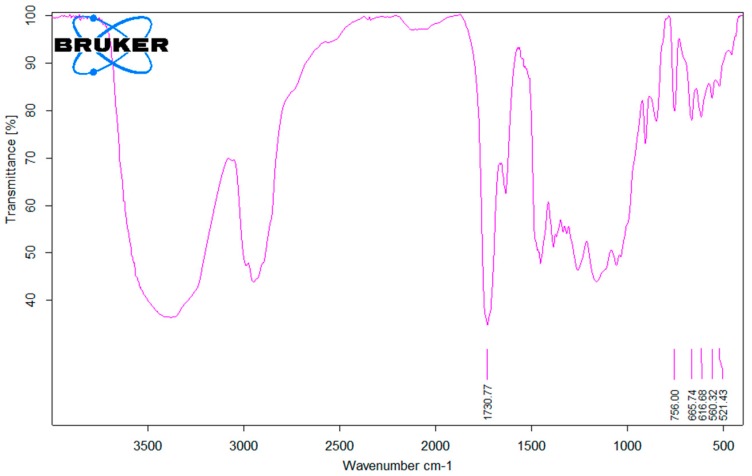
BNC-GMA-EGDMA MS = 1 FT–IR spectrum.

**Figure 13:**
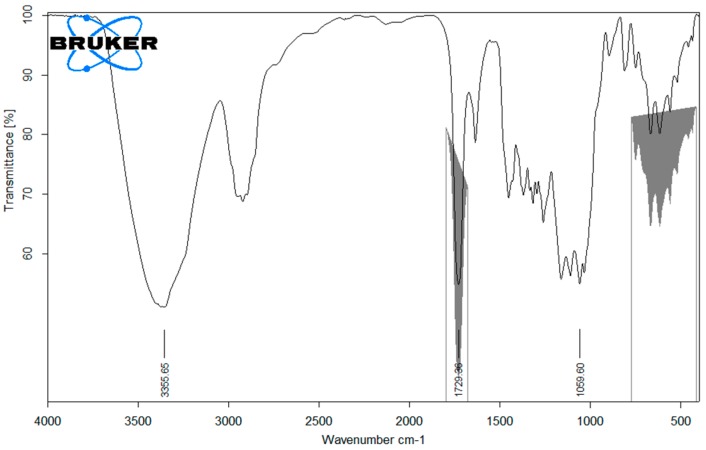
BNC-EGMA MS = 0.5 FT–IR spectrum. EGDMA ester group signal and cellulose reference signals in evidence.

BNC-GMA-EGDMA FT–IR spectra at different MS are reported in Figure 11 and Figure 12. As expected, spectra are quite similar to BNC-GMA spectra, due to the presence of the ester groups of GMA and EGDMA that are overlapped. They show also the epoxide group signals. The spectrum of BNC-EGDMA reported in Figure 13 shows only the ester group signal.

**Figure 14:**
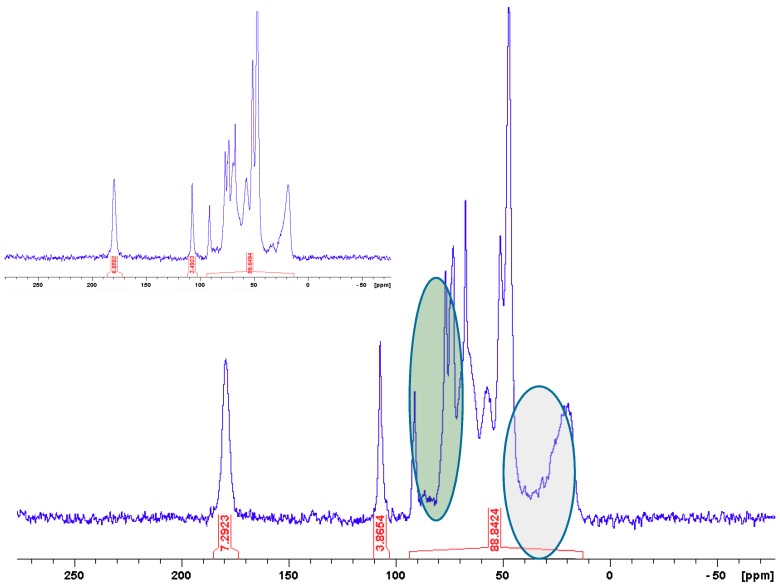
CP-MAS ^13^C NMR of BNC-GMA-EGDMA (MS >> 1)-integration Cr. I % ≈ 69. Comparison with BNC-GMA spectrum (top left) of quite the same MS. Grey background CH_3_, green background C-O.

CP-MAS ^13^C NMR high functionalized BNC-GMA-EGMA (MS >> 1) spectrum reported in Figure 14 confirms the occurrence of the GMA and EGDMA grafting by comparison with the BNC-GMA spectrum as the methacrylate residues are quite similar, but not equal, see the difference with BNC-GMA spectrum for 10–30 peaks (CH_3_ aliphatic signals) and the difference in the range 60–80 peaks (C–O signals). Also for high functionalized BNC-GMA-EGMA the crystallinity is maintained respect to the starting BNC.

### 3.2. SEM Analysis

**Figure 15:**
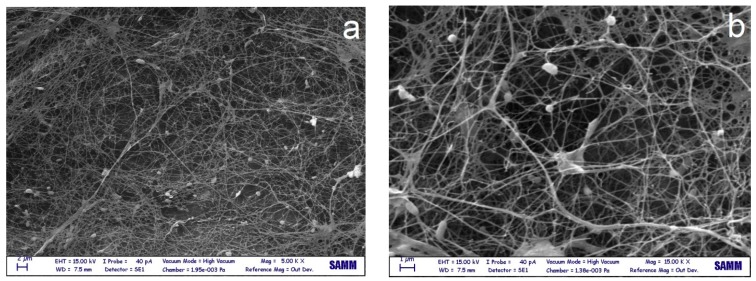

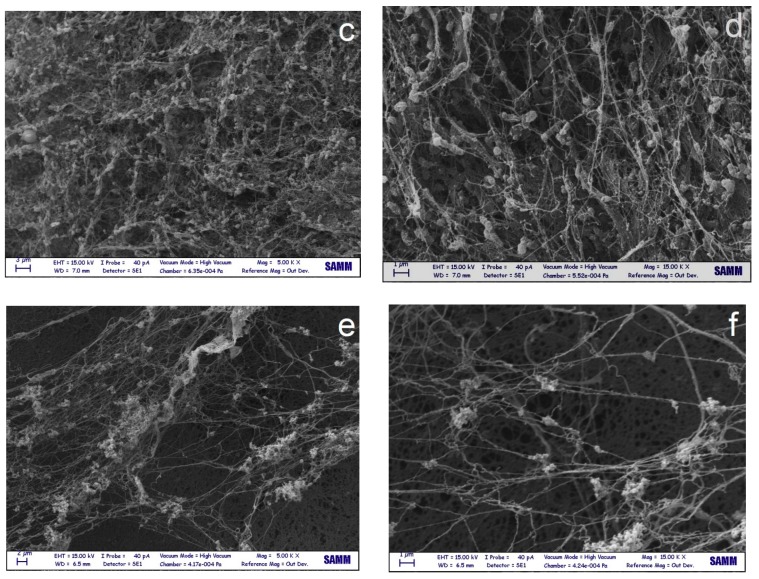
Scanning electron microscopy (SEM) micrographs of bacterial nanocellulose: (**a**,**b**) BNC as it is; (**c**,**d**) BNC-GMA (0.64 MS); (**e**,**f**) BNC-GMA-EGDMA (0.55 MS).

The SEM morphological analysis and the measurements of the fibres diameters were conducted on the following samples: BNC as it is, BNC-GMA MS = 0.64, and BNC-GMA-EGDMA MS = 0.55, see Figure 15.

The BNC as it is specimen shows the presence of a dense network of fibres with a mean diameter of 84 ± 21 nm (see Table 4). It is also noted the presence of few pseudo-spherical structure (with dimension around 1 µm) on the network.

In the BNC-GMA MS = 0.64 sample the fibres are thinner and more packed respect to the BNC as it is sample. The fibre mean diameter is 57 ± 12 nm (see Table 4). Also in this sample, the pseudo-spherical structure with dimensions of 1 µm are present and they are more numerous respect to the BNC as it is sample.

Lastly, the BNC-GMA-EGDMA MS = 0.55 sample shows the presence of thin cellulose fibres with a mean diameter of 55 ± 11 nm (see Table 4) similar to the BNC-GMA MS = 0.64 specimen. However, in this case the fibres network is less dense respect to both the samples mentioned above. Moreover, the pseudo-spherical structures with 1 µm diameter are not present, and in their place few bunches composed by spherical particles with dimension of around 200 nm can be noted on the fibre network.

### 3.3. Stress–Strain Graphic

**Figure 16:**
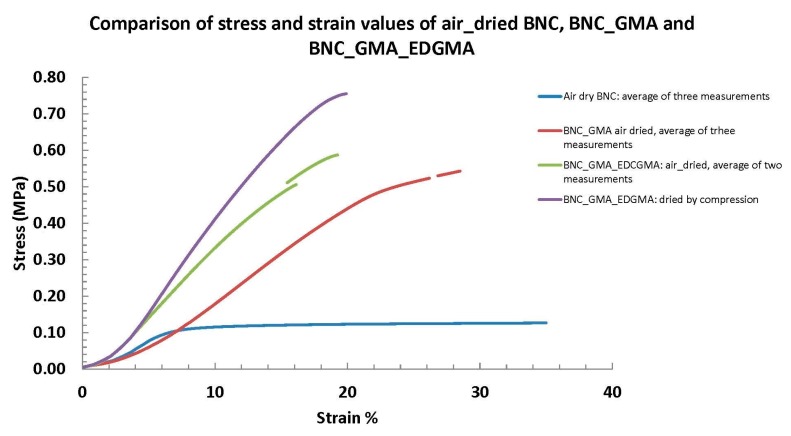
BNC (blue); BNC-GMA 0.64 MS (brown); BNC-GMA-EGDMA 0.7–1 MS (green and violet) stress and strain.

From the Figure 16 it appears that both BNC grafting and cross-linking modification enhance the mechanical resistance respect to the BNC as it is. Our strategy was not to dry the samples but only to reduce the water content with the aim of testing samples as similar as possible to the real BNC. Tested samples were left in the air for 2 h and lost the 50% of the water. In 5 h they can lose most of the water. The GMA grafting alone with MS = 0.64 significantly reinforced the fibres in the hydrogel. The grafting/cross-linking with a quite similar MS had a higher effect. The best BNC in term of resistance is the BNC-GMA-EGDMA sample with MS in the range 0.74–1 with water content reduced by compression to 20%.

### 3.4. Adsorption and Release Antibacterial Activity Test

#### 3.4.1. Adsorption and Release

The contact time between BNC and the drug solution was 24 h. Every hour the drug solution was UV analysed. No change occurred in the drug concentration. This observation allowed us to calculate the amount of drug adsorbed by the sample by diffusion from the drug solution into the hydrogel. The same drug concentration inside and outside the hydrogel was the equilibrium situation. 

**Figure 17:**
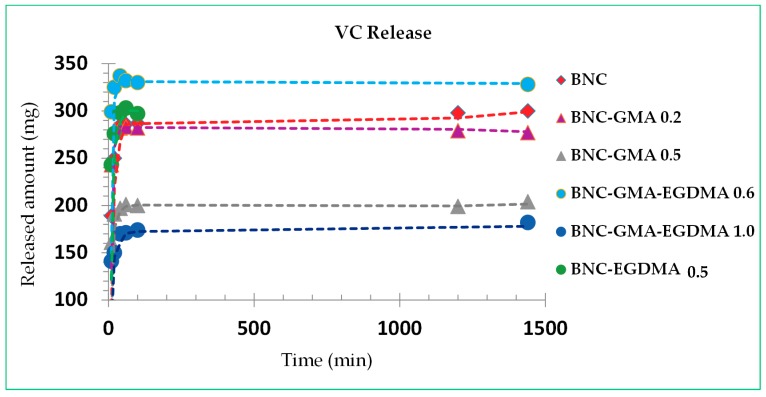
Kinetic of vancomycin (VC) release from BNC (red); BNC-GMA MS = 0.2 (violet) and MS = 0.5 (grey); BNC-EGDMA MS = 0.5 (green); BNC-GMA-EGDMA MS = 0.6 (light blue) and MS = 1.0 (blue).

**Figure 18:**
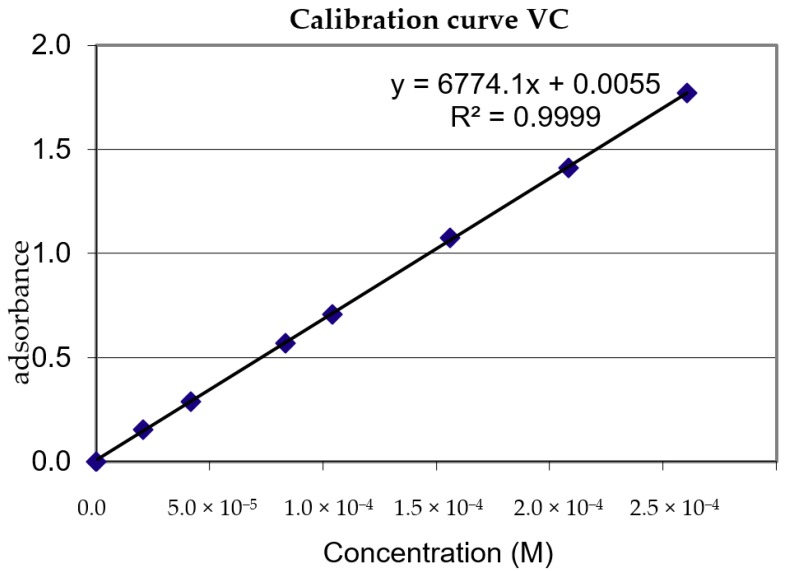
Calibration curve of VC at 272 nm, absorbance versus concentration.

In Figure 17 the amount of VC release from different BNC sample versus time measured by UV analysis at 280 nm is reported with the calibration curve in Figure 18. By elaboration on the experimental data and normalizing the value for 1 g of dry nanocellulose the equilibrium amount is 290 mg for VC, and 72 mg for CP. 

In the graphic, the amount is reported for 1 g of dry nanocellulose. In 100 min all the samples reached a horizontal asymptote. BNC amount release is near 290 mg, thus supporting our interpretation of the absorption diffusive phenomenon. BNC-GMA 0.2 MS behaviour is quite similar to BNC. The cross-linked BNC-GMA-EGDMA with MS = 1 holds half of the adsorbed VC. The same behaviour is shown by BNC-GMA 0.5. By contrast, BNC-EGDMA 0.5 behaves as BNC. BNC-GMA-EGDMA with MS = 0.6 data could be attributed to an active adsorption, but it could be also considered in the range of the experimental error, suggesting that only highly cross-linked BNC-GMA-EGDMA really holds VC.

**Figure 19:**
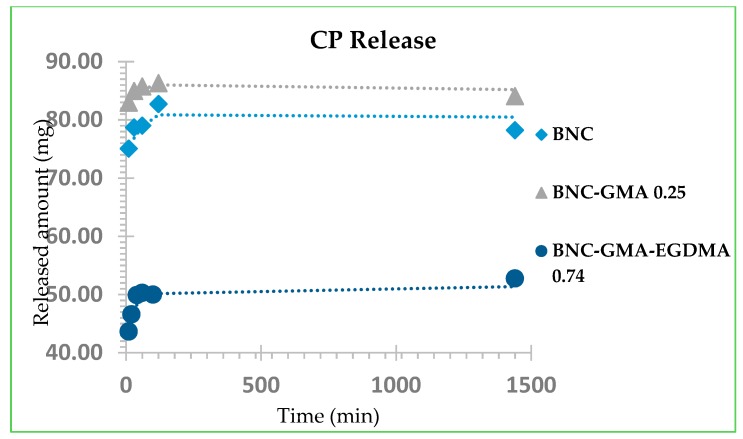
Kinetic of ciprofloxacin (CP) release from BNC (light blue); BNC-GMA MS = 0.25 (grey); BNC-EGDMA MS = 0.74 (blue).

**Figure 20:**
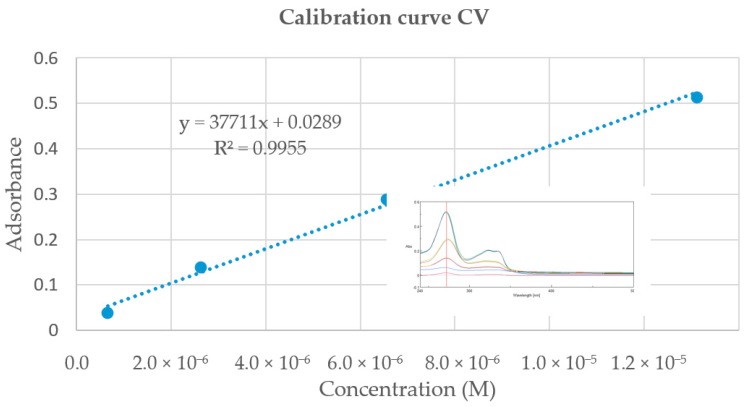
Calibration curve CP at 280 nm, absorbance versus concentration.

In Figure 19, the amount of CP release from different BNC sample versus time measured by UV analysis at 272 nm is reported, with the calibration curve in Figure 20. As with VC, in 100 min the three samples reached a horizontal asymptote. BNC amount release is near 80 mg, in good agreement with the theoretical value of 72. BNC-GMA with MS = 0.25 data could be attributed to an active CP adsorption. More evident is the behaviour of BNC-GMA-EGDMA with MS = 0.74, that holds 37% of the adsorbed CP.

#### 3.4.2. Antibacterial Activity Tests

**Figure 21:**
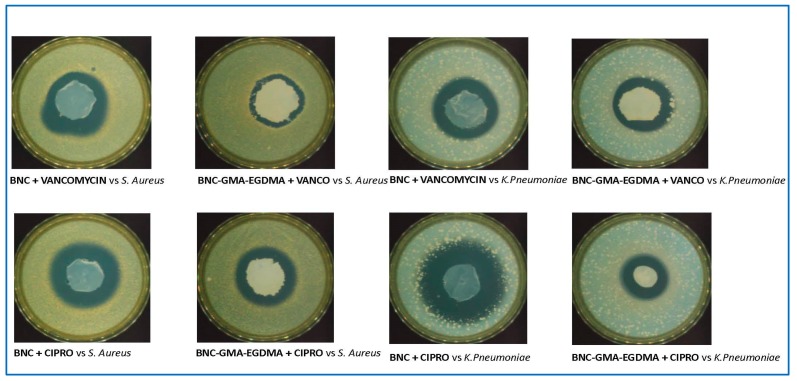
BNC and BNC-GMA-EGDMA MS = 0.8 antibacterial activity test. Left *S. aureus*, right *K. pneumoniae*. Upper VC, bottom CP.

BNC-GMA-EGDMA were used for antibacterial activity tests on the base of mechanical strength and release results. Four disks of BNC of 2.2 g were transformed in BNC-GMA-EGDMA with MS = 0.8–1, loaded with VC and CP according to the adsorption procedure and washed for 10 min in water. Four disks of BNC of 2.2 g were equally loaded with VC and CP according to the adsorption procedure and washed for 10 min in water, before the test. Every single disk was cut in four pieces and submitted to the in vitro tests described in paragraph 2. They did not undergo any degradation process. Their pictures are shown in Figure 21. The tests gave very positive results as all samples were not colonized by *Staphylococcus aureus* (*S. aureus*) and *Klebsiella pneumoniae* (*K. pneumonia*). The inhibition zone measurements are reported in Table 5. BNC zones of inhibition are larger than the corresponding BNC-GMA-EGDMA zone of inhibition. This behaviour appears in agreement with the release data and suggests the more long term antibacterial capability of BNC-GMA-EGDMA with respect to BNC.

## 4. Discussion

The study presented in this paper can be considered the first step towards a platform of new biomaterial based on BNC, built up with a method that was not yet investigated for nanocellulose. The results obtained with these studies start from the real possibility of applying to nanocellulose a process that was extensively studied for cellulose [16]. The peculiarity of this process is that it adds new properties to cellulose without modifying the original ones. The explanation for these results is the mechanism that involves two steps; the first is the generation by hydrogen abstraction of carbon centred macroradicals, sterically hindered and for this reason particularly stable. According to the polar effects involving in selective radical trapping process, these nucleophilic radicals are quickly trapped by double bonds substituted by electron withdrawing groups. Methacrylate groups are the ideal scavengers. The results of the scavenging process are the formation of a very stable C–C bond, and the maintenance of the glucose structure in the cellulose polymers that guaranties the maintenance of the cellulose properties. If many radicals are formed as the same time by a high reactive and unselective radical like OH^.^ generated by a Fenton-type process, the scavenging step endows the cellulose surface of a network of methacrylate residues that is capable of capturing molecules like a sponge [16]. In collaboration with clinics of the Humanitas Hospital in Rozzano, Milano, Italy, we demonstrated that polysaccharide materials derivatised with GMA and loaded with antibiotics have topic antibacterial activity [17,18]. The clinics made in vitro and ex-vivo tests that stated the compatibility of GMA modified gauze with biological medium and with human skin. These results support the hypothesis that the same modification on nanocellulose leads to biocompatible materials. On the other hand the biocompatibility of BNC is well-established [6] and nanocellulose materials have been recently critically reviewed for drug delivery [27]. In particular they have shown considerable potential for developing a novel generation of controlled drug delivery for different routes of administration (oral, transdermal, etc.).

The transfer of this process to BNC was satisfying. As for the grafting on cellulose, the temperature is crucial in the first step in order to maintain the radical chain, as Fe(II) is used in catalytic amount and needs to be regenerated, and also in the second step to solubilize the methacrylate monomers in water. Entry 6 in Table 1 shows that the temperature cannot be reduced from 80 °C to 60 °C even with high reagents amounts. The Fenton-type process on BNC was successfully expanded to EGDMA, affording cross-linking that was not studied on cellulose. The cross-linking was finalised to enhance the resistance of BNC and to form on BNC a network with GMA of high loading capability and of long-term antibacterial property. The main drawback is the homogeneity when the starting BNC is of the order of magnitude of more than 2 g, due to the difficulties of efficient stirring. Future research directions will be the design of a suitable device to guarantee homogeneity, scale up, and economic evaluations of the process.

It is well-known that the properties of hydrogel state appear to be absolutely suitable to develop biomaterial in biomedical applications. The purpose of this study was to use both BNC and modified BNC in the hydrogel form. By contrast with many studies on BNC, we decided not to dry and rehydrate the samples, to reduce the step numbers to reach the final products. The percentage of nanocellulose in our modified BNCs increased in agreement with the MS, but our modified BNCs remained in the hydrogel state if the grafting and cross-linking reaction conditions were carefully controlled to not overcome MS = 1. At high MS, modified BNC became quite solid, due to water elimination that reached the 50%. Solid modified BNCs are not the achievement of this study, but they could be further developed as solid functionalised nanocelluloses of high purity. 

SEM images and mechanical tests we presented cannot be compared with literature data that are mainly produced on dry samples. However mechanical tests of our modified BNC are very encouraging with respect to BNC as it is, in terms of resistance to the stress and strain. As expected, the cross-linking in particular has a positive effect on mechanical resistance, supporting the aim of our studies to make BNC cross-linked. SEM images are very interesting as it seems that GMA alone forms a dense thick network, while GMA-EGDMA network appears less dense. The density of the GMA network certainly helps the drug to be captured. GMA-EGDMA network is less dense, but due to the cross-linking is more rigid. This could induce a more stable electrostatic interaction between the GMA appendages and the drug. From a thermodynamic point of view we could argue that the loading enthalpy factors are more favourable for GMA entrapped in the cross-linking than for mobile GMA residues. At the same time, the BNC network was maintained. The observed pseudo-spherical structures could be explained by the formation of small pieces of cotton. It will be necessary to reconsider starting BNC preparation to avoid them.

It is very intriguing to comment on the crystallinity of the modified BNC that is maintained also at high MS equal to the BNC as it is. This behaviour confirms that the Fenton-type process we developed respects the morphology not only for cellulose, but also for nanocellulose. However, in our opinion the mechanism is not exactly the same. In the case of cellulose the functionalization occurs on the surface and in the bulk, while in the case of BNC it occurs on the fibre surface and does not modify at all the fibre itself.

To make the BNC bioactive we decided to use real antibiotics, with the idea that although antibiotics can develop resistance, they are the most potent tools we have to fight against infection. Hospital infections, surgical complications infections and wound infections are a tremendous problem for all of us and represent a relevant social cost, too.

BNC as it is appears suitable to host antibiotics like VC and CP, whose activities are well-established. VS is especially important in its use in the hospital.

Modified BNCs loaded with VC and CP can be developed as antibacterial materials. This conclusion is supported not only by adsorption release data collected in the laboratory, but also by the antibacterial activities tests entrusted to the expert Centrocot staff that use two very dangerous bacteria like S.A. and K.P. BNC-GMA and BNC-GMA-EGDMA are not as available as BNC. However their properties justify the effort to prepare them. First of all, the mechanical properties are relevant. GMA appendages alone and associated with EGDMA cross-linking are capable of enhancing the BNC resistance. But likewise important is their interaction with the antibiotics. Efficient loading and holding depend on MS. BNC-GMA are efficient in the loading and holding of VC and CP, similarly to GMA modified cellulose. The only cross-linking with EGDMA does not seem efficient enough to hold drugs. The grafting and cross-linking are responsible for the most efficient holding capability. BNC-GMA-EGDMA behaviour suggests that there is a synergy of EGDMA and GMA to efficiently hold the drug. The synergy of EGDMA and GMA can be interpreted in the sense that GMA appendages create a network on a single cellulose chain and that EGDMA creates a network among different cellulose chains. Thus, BNC-GMA and BNC-GMA-EGDMA could open the way to a new kind of BNC, suitable to modulate the drug-delivery phenomenon in biomedical applications.

## Figures and Tables

**Table 1:** Fenton-type glycidylmethacrylate (GMA) grafting. Relationship between reagent molar ratio and bacterial nanocellulose-GMA methacrylate residue per glucose unit (BNC-GMA MS).

Entry	Glucose (mmol)	FeSO_4_ (mmol)	H_2_O_2_ (mmol)	GMA (mmol)	MS
1	1	6.50 × 10^−1^	800	220	> 3
2	1	3.25 × 10^−1^	400	110	2.8
3	1	1.63 × 10^−1^	200	55	1.8
4	1	8.1 × 10^−2^	100	30	0.8
5	1	4.0 × 10^−2^	50	14	≈0.0
6 ^1^	1	3.20 × 10^−1^	920	610	0

^1^ Reaction temperature 60 °C.

**Table 2:** Fenton-type GMA grafting in function of the reagent amount.

Entry ^1^	BNC/Glucose	FeSO_4_^2^	H_2_O_2_^3^	GMA	MS
1	2.1 g	0.10 mL	0.66 mL	0.24 mL	0.12–0.32
	(1 mol)	(0.04 mol)	(49.5 mol)	(13.7 mol)	
2	2.1 g	0.16 mL	1.00 mL	0.35 mL	≈0.25
	(1 mol)	(0.06 mol)	(74.36 mol)	(20.53 mol)	
3	2.1	0.21	1.32	0.47	0.3–0.7
	(1 mol)	(0.08 mol)	(99.13 mol)	(27.37 mol)	

^1^ In bracket, the molar ratios normalized on 1 mmol of glucose unit. ^2^ 0.05 M FeSO_4_∙7H_2_O solution. ^3^ H_2_O_2_ solution 30% (*w*/*w*).

**Table 3:** Fenton-type GMA grafting and ethylene glycol dimethacrylate (EGDMA) cross-linking in function of the reagent amount.

Entry ^1^	BNC/Glucose	FeSO_4_	H_2_O_2_	GMA	EGDMA	MS
1	2.2 g	0.16 mL	1.0 mL	0.26 mL	0.156 mL	0.6–0.74
	(1 mol)	(0.06 mol)	(74.36 mol)	(14.37 mol)	(6.2 mol)	
2 ^2^	2.2 g	0.16 mL	1.0 mL	0.26 mL	0.156 mL	≈1
	(1 mol)	(0.06 mol)	(74.36 mol)	(14.37 mol)	(6.2 mol)	
3	0.534	0.04 mL	0.308 mL	0	0.128 mL	0.55
	(1 mol)	(0.06 mol)	(74.36 mol)	0	(20.53)	

^1^ In bracket, the molar ratios normalized on 1 mmol of glucose unit. ^2^ Reaction times 1 h.

**Table 4:** Results from the dimensional analysis of BNC fibres displayed in the SEM micrographs.

Sample	BNC	BNC-GMA 0.64	BNC-GMA-EGDMA 0.55
mean fibres diameters	84 ± 21 nm	57 ± 12 nm	55 ± 11 nm

**Table 5:** BNC and BNC-GMA-EGDMA antibacterial activity test, inhibition zone in mm in Figure 21.

Entry	Sample	VC	CP	*S. Aureus*	*K. Pneumoniae*	MS
1	BNC-GMA-EGDMA	x	-	4	-	0.8–1.0
2	BNC	x	-	8	-	-
3	BNC-GMA-EGDMA	-	x	8	-	0.8–1.0
4	BNC	-	x	10		-
5	BNC-GMA-EGDMA	x	-	-	5	0.8–1.0
6	BNC	x	-	-	7	-
7	BNC-GMA-EGDMA	-	x	-	11	0.8–1.0
8	BNC	-	x	-	15	-
